# Aryl-Substituted Dihydro-Pyrimidines Effecting Kinesin Eg5 as Novel Approach for Cancer Treatment

**DOI:** 10.3390/molecules30153256

**Published:** 2025-08-03

**Authors:** Dialekti Chlorou, Eleni Pontiki

**Affiliations:** Laboratory of Pharmaceutical Chemistry, Faculty of Health Sciences, School of Pharmacy, Aristotle University of Thessaloniki, 54124 Thessaloniki, Greece; dchloro@pharm.auth.gr

**Keywords:** Κinesin Eg5, dihydropyrimidinones, aryl-DHPM, anticancer, monastrol, cell lines

## Abstract

Cancer is one of the most lethal diseases of this century. Unfortunately, many anticancer agents have harsh side effects or fail to work against cancer any longer due to tolerance. Dihydropyrimidinones are promising structures containing a pyrimidine ring. Targeting Eg5 is their most well-known activity. Inhibition of this enzyme gives them the privilege of strong cytotoxic activity with less side effects. Phenyl ring is a group that can be found in the majority of organic molecules and possesses preferable pharmacokinetic and pharmacodynamic characteristics. This review studies DHPM derivatives that are substituted with a phenyl ring and possess antiproliferative ability by inhibiting Eg5. The compounds are able to inhibit different cancer cell lines, and some are more potent than the standard drug. The biological results are in accordance with the docking studies.

## 1. Introduction

Cancer is one of the two diseases that are known for their high incidence and mortality rate. The other one is cardiovascular diseases, but it is said that, in the future, cancer will surpass them as the leading cause of death. Cancer is a complex disease that can be caused by numerous factors (genetic mutations, chemicals, infections, etc.), characterized by uncontrollable cell proliferation and invasion to healthy tissues. Despite the fact that many attempts have been made to defeat cancer, no permanent cure has been found yet. The anticancer agents that are being used currently interrupt the cell cycle through affecting tubulin, vinca alkaloids through its polymerization, and taxanes through its stabilization. Unfortunately, due to the importance of tubulin for the maintenance of cytoskeleton functions and intracellular transportation, these compounds have harsh side effects to the nervous system like neutropenia, peripheral neuropathy, and severe myelosuppression. In addition to this, there is the resistance that is developed by cancer cells. Hence, the need for anticancer agents that are safe and efficient is imperative [1,2,3].

### 1.1. Kinesin Eg5 Implication in Cancer Progression

**Figure 1:**
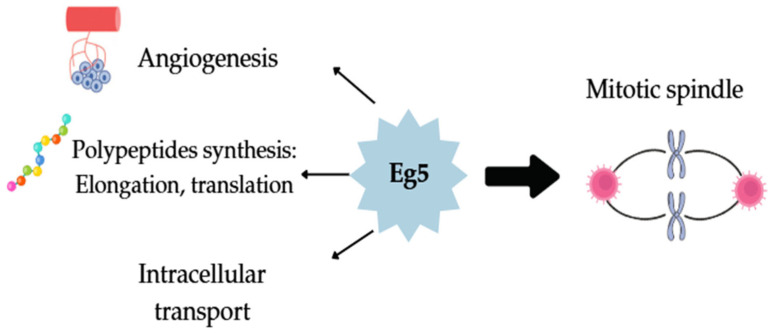
Eg5 role in cancer with mitotic implication being the most important.

One phenomenon that is closely related to cancer progression is the overexpression of kinesin Eg5. This protein can be detected in higher amounts in cancer cells compared with normal ones, and, therefore, it is also used as a biomarker or a therapeutic target. Eg5 was the first known protein to be coded by an mRNA, and, along with the results from antibodies that can recognize short peptides with conserved sequences, it was confirmed that Eg5 is a member of the kinesin motor proteins superfamily. It is also known as Kinesin Spindle Protein (KSP) or KIF11 (which is the gene that encodes Eg5). Kinesin Eg5 is homotetrameric motor protein, which, as any other kinesin, contains the most crucial parts for its function, like the nucleotide-binding and the microtubule-binding site, to the motor domain. This is a highly conserved domain with topological similarity to other kinesins; the only difference is that the L5 loop of Eg5 is the longest. The L5 loop is located in the a-helix and is the place where molecules like monastrol react. Eg5′s proper function plays a role in numerous functions of a cell. For example, it contributes to the synthesis of proteins (participates in the elongation and the termination of translation) and to their transportation (from the trans-Golgi to the cell surface). What is more is that it supports angiogenesis as well as the axonal and dendritic transport of postmitotic neurons. Nonetheless, its most well-known ability is related to the mitotic spindle (Figure 1). To be more specific, using the hydrolysis of ATP as an energy source, it moves on the microtubules, and, through this, it separates the two centrosomes and the bipolar formation. Normally, it is inactive, but during prophase and metaphase, it converts to its active form and increases in number. For its activation, a series of phosphorylations and deacetylations on different sites of its structure are essential with the phosphorylation of its C-terminus by Cdk1 being the most important. Kinesin’s Eg5 functionality can show whether bipolar spindle formation can be successful or not. In case Eg5 is unable to properly function and a monoester gets formed, then the path to programmed cell death will be triggered. As a result, Eg5 can be a potential target for treating cancer [4,5,6].

### 1.2. Dihydropyrimidinones

**Figure 2:**
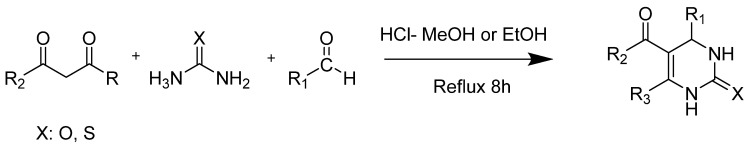
General synthesis of DHPM.

**Figure 3:**
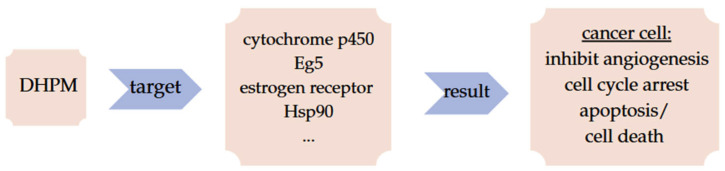
DHPM possible targets.

Heterocyclic compounds are involved in different fields like agrochemical, veterinary, and medicinal chemistry. Especially nitrogen-containing compounds are known for having diverse medicinal properties. Pyrimidines are the most well-known structure of this category. Pyrimidines are structural components of nucleic acid bases, like thymine, uracil, and cytosine. Being hydrophobic at the cell’s neutral pH, they present low solubility in water. Should the pH change, and become either alkalic or acidic, they will convert to their ionized form increasing their solubility. Dihydropyrimidinones (DHPMs) are the most significant class of pyrimidines. They are doubly unsaturated six-membered rings that have two nitrogen atoms at positions 1 and 3. They are advantageous structures since their chemistry and synthesis (Figure 2) are quite simple, and they can be the basis of many compounds in medicinal chemistry. Apart from these abilities, dihydropyrimidinones’ numerous pharmaceutical properties make them attractive too. They can aim at multiple targets, e.g., enzymes like cytochrome p450 and receptors such as estrogen, that are essential for the development and progression of cancer. Depending on their target, dihydropyrimidinones can promote antiangiogenesis. They inhibit Eg5 or Hsp90 and, as a result, cause cell cycle arrest or even obstruct mitotic saft, leading to apoptosis or cell death. As a result, they can combat a diversity of malignancies such as breast and blood cancer (Figure 3) [7,8,9].

### 1.3. DHPM and Eg5

**Figure 4:**
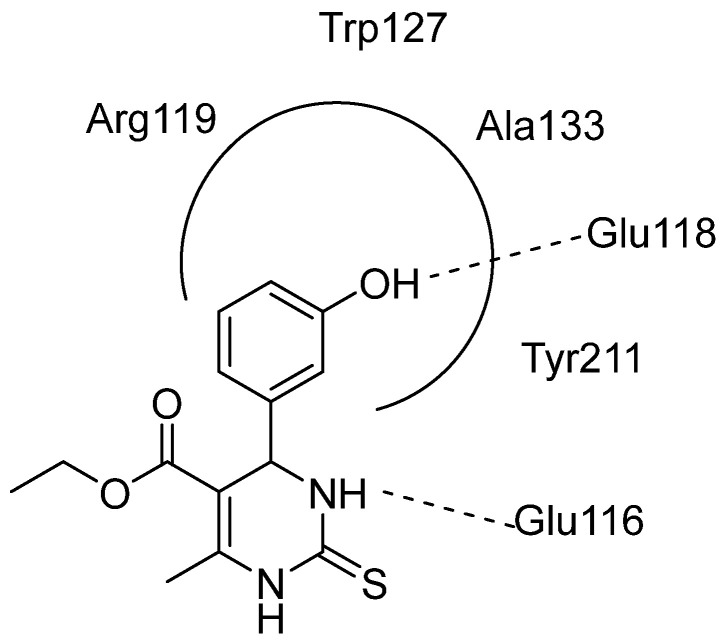
Monastrol’s putative interactions with Eg5.

DHPMs are well known for their ability to inhibit Eg5. The first and most popular DHPM derivative to be reported, which bears this property, is monastrol. Monastrol is an FDA-approved compound that generates monoastral spindles that are surrounded by chromosomes and cause cell death. What is noteworthy is that it does not affect the nervous system, since it shows no neuronal cytotoxicity. Its mechanism of action is quite simple; it allosterically binds to Eg5 making a ternary complex with the enzyme and ADP. This causes changes to the enzyme structure preventing the binding of the ATP to the active site, and, thus, it stops Eg5 from binding to the microtubules. Hydrophobic interactions can be observed between monastrol and amino acids of Eg5 such as Glu116, Ile136, Pro137, Tyr211, Leu214, and Ala218. The hydroxyphenyl group can be found in a hydrophobic pocket of Eg5 interacting with Arg119, Trp127, Ala133, and Tyr211. Additionally, hydrogen bonds between monastrol’s N atom with Glu116 and monastrol’s hydroxyphenyl oxygen with Glu118 are developed, respectively (Figure 4). The same interactions can be observed with DHPM derivatives. Despite monastrol’s activity, it presents poor pharmacokinetic characteristics, making it an unsuitable drug candidate. However, it is the lead compound for the synthesis of many other DHPMs that are Eg5 inhibitors. Over the years, multiple DHPMs with different structures were created bearing the phenyl group as the most common substituent enhancing the inhibitory activity [10,11,12,13].

The aim of this review is to study recent literature from 2018 to 2025 concerning aryl-DHPM as KSP inhibitors highlighting the most favorable structural characteristics, helping the synthesis of selective, efficient, and safe anticancer agents.

## 2. Novel Aryl-DHPM Derivatives

**Figure 5:**
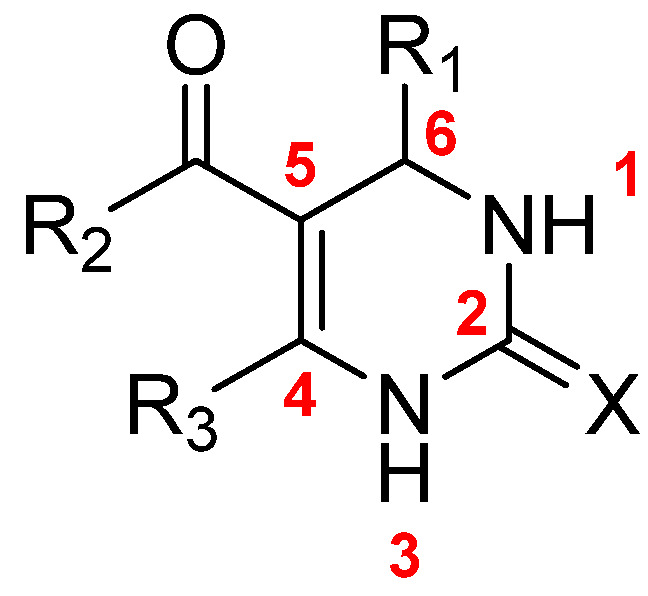
General structure of DHPM.

For practical reasons, the structures will be categorized based on the position of the phenyl ring on DHPMs (Figure 5).

### 2.1. N3-Substituted Derivatives

**Figure 6:**
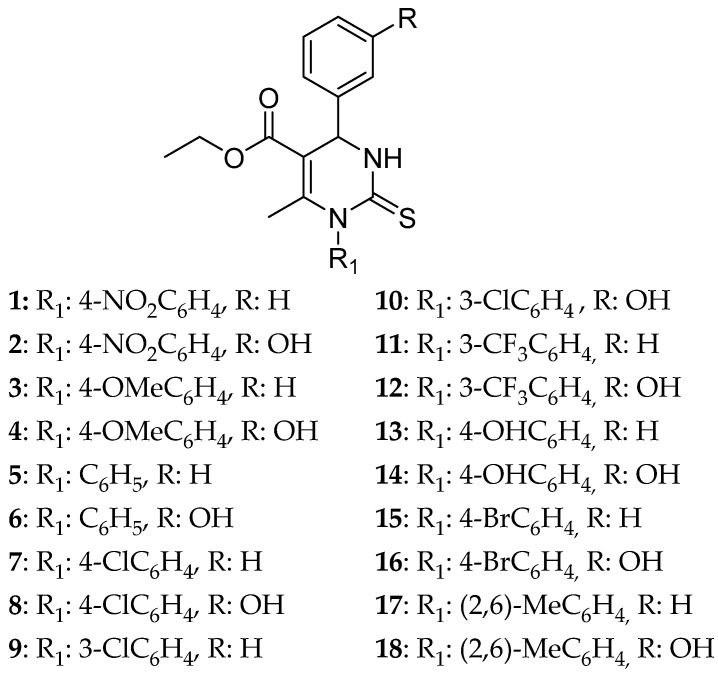
Series of substituted biphenyl DHPM synthesized by Goncalves et al. [2]. The most promising ones are compounds **8, 12**, and **16**.

Goncalves et al. [2] emphasize the ability of monastrol, a DHPM structure, to inhibit kinase Eg5 and making a most promising anticancer agent. They studied the role that the substitution on N3 plays in the anticancer activity of a DHPM structure. They used a library of already published DHPM [14] and studied a series of 18 monastrol analogues (entries 1–18) (Figure 6) intending to find the substituents enhancing the anticancer activity of DHPMs. The novel derivatives have been evaluated for their cytotoxic effect on glioma cell lines U-138 using monastrol as the standard drug. From the 18 derivatives, only 3 compounds have shown better activity than the reference drug. From screening on glioma cell lines, the most potent derivatives were compounds **8**, **12**, and **16** presenting 80%, 82%, and 81% inhibition, respectively, in a concentration of 150 μΜ.

It can be concluded that N3 phenyl substitution generally increases the cytotoxic effect compared with monastrol. To be more specific, electronegative groups at the meta and para position improve their ability. This can be attributed to the bulky atoms filling the P3 pocket of the enzyme Eg5. In addition to this substitution, the -OH group on the para position of the phenyl ring connected to C6 increases the anticancer ability of a compound. These three compounds were further tested for their anticancer activity on bladder cancer cell line T24. Again, in this cell line, these three derivatives proved to be stronger than the standard drug. What was noteworthy was compound **16** that possessed an IC_50_ = 18.52 ± 7.83 μM, while monastrol’s was IC_50_ = 262.66 ± 61.63 μM. QSAR studies were in accordance with these results showing that strong cytotoxicity is closely related to bulky and electronegative substituents. The three most efficient derivatives bear a 3OH-phenyl group at C6 and a phenyl ring substituted with an electron withdrawing group at N3. Compounds with no substitution on the phenyl group or 4-OCH_3_ showed the lowest cytotoxic effect being inactive in terms of QSAR studies. It seems that substituents of the N3-phenyl ring play an important role. They can be categorized from the less active to the strongest one: 4-OCH_3_ ≈ H ≈ 4 NO_2_ < 2,6-CH_3_ < 4-OH < 3-Cl < 4-Cl ≈ 3-CF_3_ ≈ 4-Br. The molecules have been proven to be safe for the normal cells from in vivo studies on C. elegans with LC_50_ higher than the needed concentration for cell death, 7.02 ± 1.03 and 4.45 ± 0.99 mM for compounds **12** and **16**, respectively, and 2.93 ± 0.25 mM for compound **8**. Additionally, DCF-DA assay was conducted on C. elegans where compounds **12** and **16** showed antioxidant activity; compound **12** was the only one that can inhibit the production of ROS in vivo. To further confirm the results regarding the antioxidant ability of a structure, DPPH assay was conducted. Being an in vitro experiment, the specificity is low, and the results cannot be affected by outer factors like when in an in vivo assay.

### 2.2. C5-Substituted Derivatives/R2 Substituted

**Figure 7:**
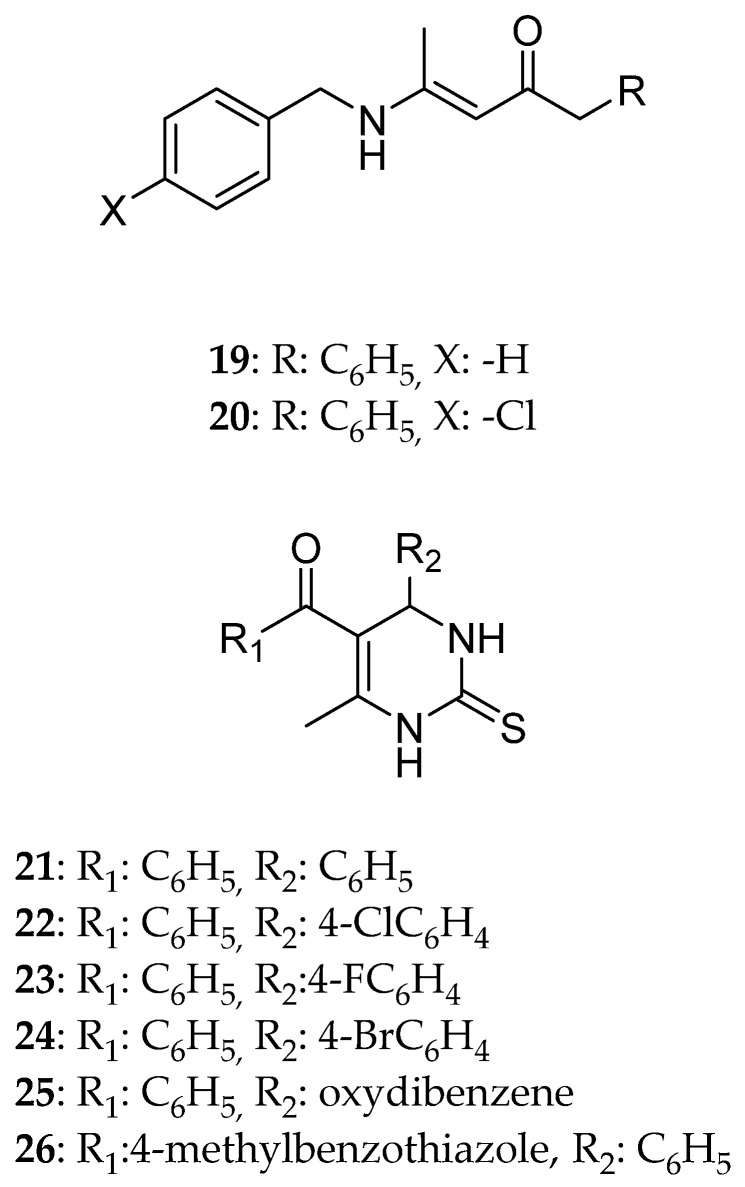
The acyclic enamino amides and dihydropyrimidinethiones that Bohlooli et al. [15] synthesized; the most potent compounds were **23** and **25**.

Bohlooli et al. [15]. synthesized a series of dihydropyrimidinethiones (DHPMTs) and acyclic enamino amides by altering the structure of monastrol, which is a well-known Eg5 inhibitor (Figure 7). The purpose was to possess stronger anticancer effect. DHPMTs’ cytotoxic ability was tested on human gastric (AGS), liver (Hep-G2), and breast (MCF-7) cancer cell lines. Concerning Hep-G2 and MCF-7 cell lines, none of the compounds presented better activity than the standard drug. However, on the AGS cell line assay, structure **23** showed an activity of IC_50_ = 9.9 ± 0.3 μΜ, stronger than cisplatin (IC_50_ = 11.4 ± 2.9 μΜ), which was used as the reference drug. In general, the two most efficient molecules are compound **23** with IC_50_ 9.9 ± 0.3, 15.2 ± 0.4, and 40.5 ± 1.0 μΜ for AGS, Hep-G2, MCF-7, respectively, and compound **25** with 23.8 ± 1.1, 25.9 ± 7.2, and 94.2 ± 7.9 μΜ, respectively. Based on the results, it can be concluded that meta substitution with an electron withdrawing group enhances the cytotoxic activity of a compound. This can be probably attributed to the H-bonds developed with Eg5. This can be improved with bulky amide substituents, but very bulky substitution may have the opposite result due to steric hindrance. Additionally, the phenyl group that replaces the ethyl group is responsible for hydrophobic interactions with Eg5. What is noteworthy is that the oxo derivatives were less effective than the sulfur ones, which may be due to the antiproliferative ability that the latter one has.

**Figure 8:**
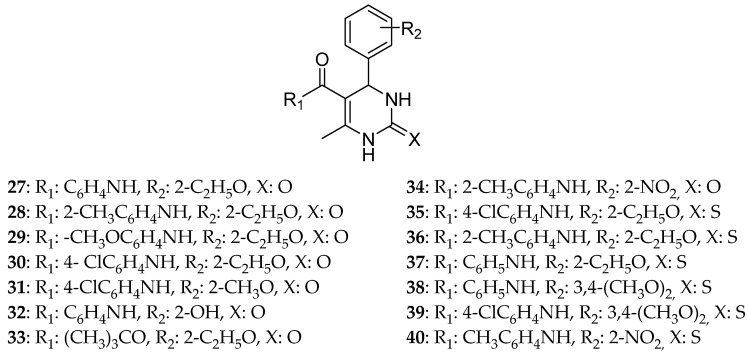
DHPM substituted with benzene ring that Jiang et al. synthesized [3]. Compound **35** is the most potent one within the series.

**Figure 9:**
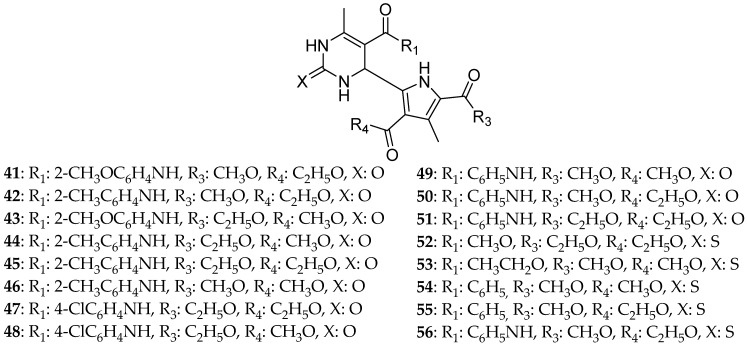
DHPM derivatives substituted with a pyrrole ring that Jiang et al. synthesized [3].

According to Jiang et al. [3], the acyl oxygen and secondary amino hydrogen atom of a dihydropyrimidine are essential for Eg5 inhibition since these two atoms are capable of forming three hydrogen bonds with amino acids of the active site. They used solvothermal synthesis, a common synthetic method in inorganic chemistry, to produce DHPM derivatives with very good yields higher than 70%. The derivatives where substituted with either a benzene ring (14 compounds) (Figure 8) or a pyrrole ring (16 compounds) (Figure 9). Firstly, the molecules were tested for their ability to inhibit Eg5, especially as ATPase inhibitors. Nine compounds were able to present results in micromolar range concentration with **35** being the strongest one with an activity of 30.25 μΜ and **54** the less active of all with an ATPase activity of 71.86 μΜ, even lower than the one of monastrol 51.74 μΜ. When it comes to the cytotoxic ability of the compounds, the most potent one of all was **35**, a DHPM containing a benzene ring, with IC_50_ 65.80 μΜ (Caco-2 cell), 75.59 μΜ (Hela cell), and 103.21 μΜ (T24 cell). It is worth mentioning that this molecule is a mixed inhibitor with Ki = 15.73 μΜ. To sum up, **35** is a more efficient inhibitor than monastrol for HeLa and Caco-2 cancer cell lines and causes an adequate inhibition of T24 (the cell line of bladder carcinoma). Its cytotoxic effect toward the murine fibroblast L929 cell line was studied showing a moderate cytotoxicity of 85.97 μΜ in a dose-dependent manner. These findings were consistent with the docking studies where **35** was surrounded by active amino acids forming three hydrogen bonds with Asp187, Lys260, and Val194 and possibly having aromatic interactions with Asp322. These results showed that compound **35** presents stronger interactions with Eg5 than monastrol. It is also concluded that the oxo derivatives did not show any anticancer effect, whereas the thio ones had adequate, with **35** being stronger than monastrol.

**Figure 10:**
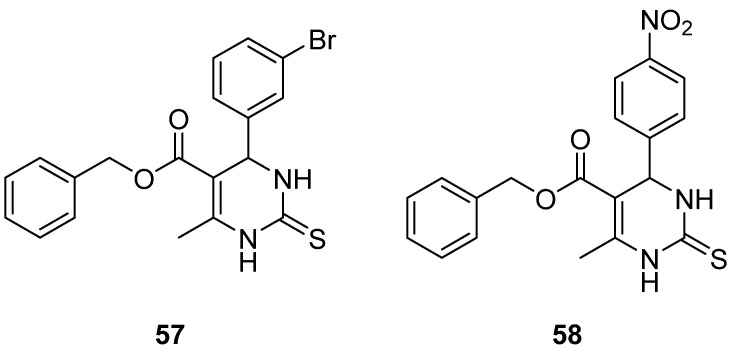
DHPM derivatives studied by Prasad et al. [9].

Prasad et al. [9]. gathered a diversity of DHPM known for their ability to inhibit Eg5 and can be used to fight breast cancer and performed structure–activity relationships (SARs) on them (Figure 10). Compounds **57** and **58,** which were synthesized by safari [1], developing hydrophobic and hydrogen interactions were quite strong against breast, liver, and lung cancer lines. Their IC_50_ were for compound **57**: 74.28 ± 8.81, 74.01 ± 5.39, and 45.85 ± 1.54 μΜ, against MCF-7, HepG3, and A549, respectively; and for compound **58**: 65.54 ± 9.06, 73.71 ± 4.26, and 43.97 ± 0.45 μΜ, respectively. From SARs, it seems that at C-6, electron withdrawing groups at the para position enhance cytotoxic activity; however, at C-5, the presence of a phenyl ring at the ester’s position reduces the anticancer ability. Lastly, the thiocarbonyl derivative was proved to be more effective than the carbonyl one.

**Figure 11:**
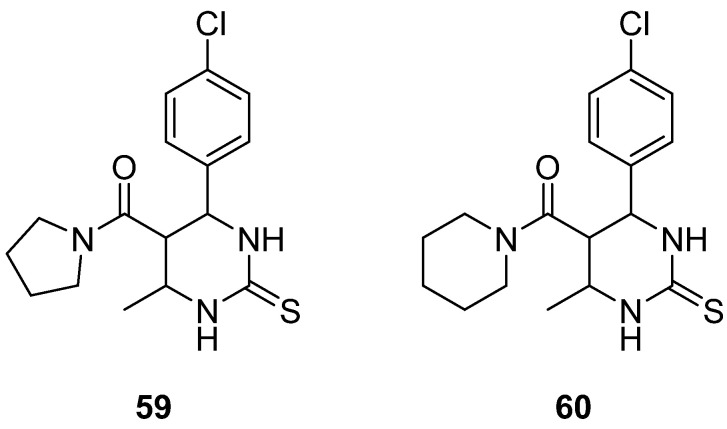
Pyrrolidine and pyridine substituted DHPM studied by Prasad et al. [9].

Regarding HepG2 and HeLa cell line inhibition, structures **59** and **60** (Figure 11) showed a moderate result, with IC_50_ 124 and 187 μΜ for **59** and 120 and 217 μΜ for **60**, respectively. Replacing the proton with -F at the p-position did not affect the general anticancer potency but increased the inhibition of cell proliferation. The absence of a hydrogen bond on the phenyl ring results in the removal of hydrogen bonds with the amino acid Glu118, which appears to be important for the anticancer activity.

### 2.3. C6-Substituted Derivatives

**Figure 12:**
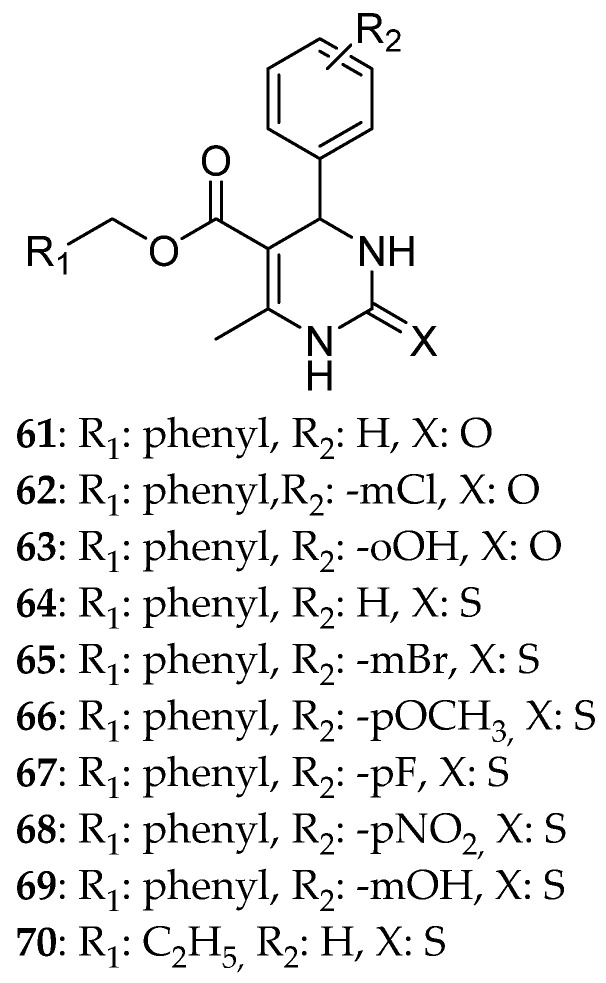
6-Methyl-2-thioxo-4-phenyl-3,4-dihydropyrimidine-5-benzoate derivatives synthesized by Safari et al. Compound **68** is the most potent within the series.

Safari et al. [1] synthesized a series of 6-methyl-2-thioxo-4-phenyl-3,4- dihydropyrimidine-5-benzoate derivatives (Figure 12) so as to create anticancer agents that will be able to inhibit Eg5. This is based on the fact that the first molecule to be observed inhibiting Eg5 making it an advantageous structure is a DHPM called monastrol. The cytotoxic ability of the compounds was tested on different cancer cell lines. Between the 10 compounds, the most effective was compound **68** with IC_50_ 65.54, 73.71, and 43.97 μM against MCF-7, HepG-2, and A549, respectively. However, its activity did not surpass the one of doxorubicin, the reference drug. Concerning their safety, normal Huvec cell line was used; all the molecules showed low cytotoxicity. It was noticed that, generally, all the molecules were more effective against the A549 cell line compared with the other ones and that a change in the substitution of the phenyl ring can drastically change the cytotoxic activity. Substitution at the ortho- and meta- position reduced the compound’s anticancer effect in comparison with the para- one, which increased it. For an even better outcome, an electron withdrawing group can be added to the para- position of the phenyl ring. The presence of fluorine in the same position just preserved the anticancer ability of the molecule. Comparing the different ways of substitution on the meta- position, the presence of Br- showed better results than the one with OH-. The interesting fact is that the derivatives with a phenyl ring instead of ethyl ester at C5- were more efficacious than the other ones. Again, thio- derivatives possessed stronger cytotoxic effect than the other ones probably due to the lipophilicity of the first one. Hence, it can be concluded that the two features that can determine the cytotoxicity of a molecule are lipophilicity and electron withdrawing groups. These results matched the docking studies. The most favorable interactions are H-bonds with amino acids Glu116 and Glu118. Compound **68** presented a binding score of −10.31 kcal/mol to Eg5. Docking studies revealed that **68** can possibly interact with Mg^2+^ located at the active site of Eg5. Hydrophobic interactions with the above Glu acids were detected. Meanwhile, other compounds like **64** formed hydrogen bonds with Glu118 and hydrophobic interactions with the amino acids of the active site. Compounds **62**, **67**, and **69** were the ones with the lowest free binding energy probably due to the presence of bulky substituents, causing steric hindrance. From all of the above, it can be concluded that the development of a bond with Mg and the simultaneous interaction of the molecule with both Glu116 and Glu118 can be essential for a strong inhibitory ability.

**Figure 13:**
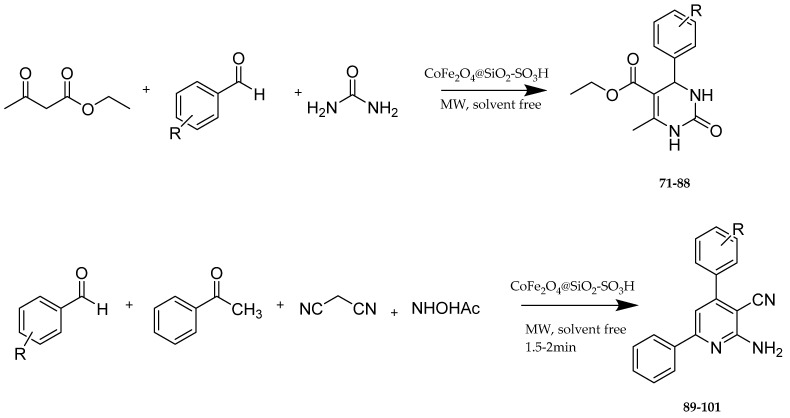
Synthetic procedure of **89-101** by Hosseinzadeh et al. [11].

**Figure 14:**
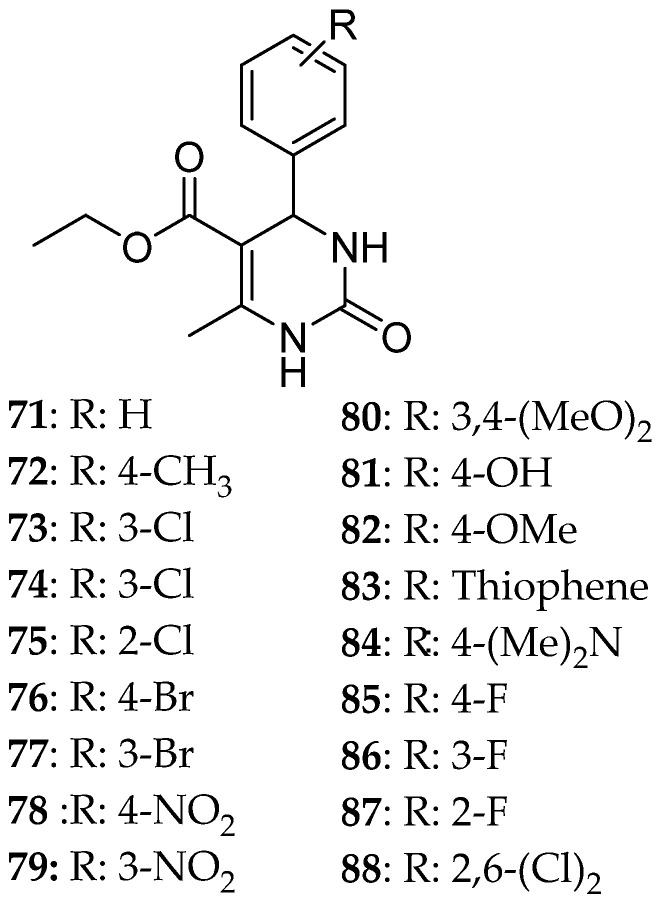
DHPM synthesized by Hosseinzadeh et al. [11]. The most potent ones are **80**, **83**, and **88**.

**Figure 15:**
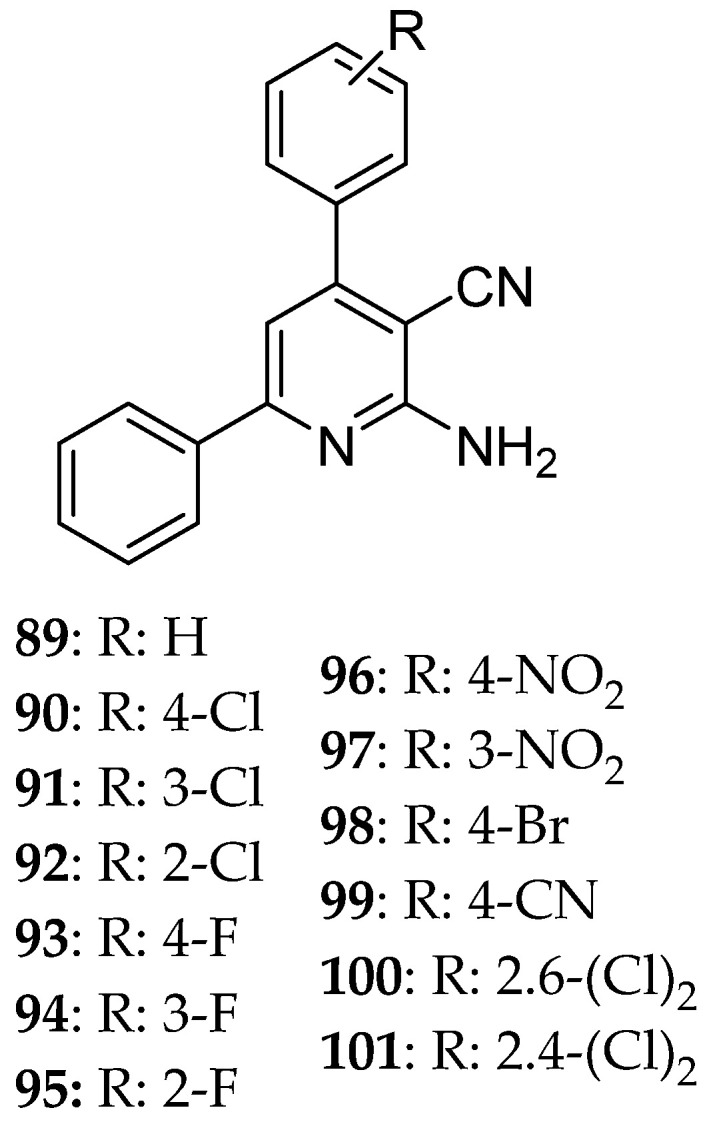
Pyridine derivatives synthesized by Hosseinzadeh et al. [11].

Hosseinzadeh et al. [11] express the major role that Eg5 plays in the development and progression of cancer, which makes it a desirable target for combating cancer. Monastrol’s ability to inhibit this enzyme is already known. They have applied the following synthetic procedure (Figure 13) for the synthesis of DHPM (Figure 14) and pyridine derivatives (Figure 15), structures of which are similar to the one of monastrol. To improve the reaction’s condition and yield, they applied SO_3_H-substituted silica-coated cobalt-based magnetic nanoparticles as a catalyst and microwave radiation for a faster reaction with high yield and without the need for a solvent (Figure 13). Then, the newly synthesized compounds were tested for their ability to inhibit the proliferation of three cell lines MCF-7, AGS (cancer cells), and HEK293 (normal cells) that was a result due to their ability as Eg5 inhibitors. The results of preliminary screenings showed that the resulting molecules will be highly cytotoxic and safer (low cytotoxic activity against normal cells). The DHPM derivatives were more efficacious than the pyridine ones. Two structures had quite promising results, **88** and **80**. After 48 hr of treating AGS and MCF-7 cells, **88** was able to cause cell death to over 50% of AGS cancer cell lines and, together with **80**, showed a remarkable cytotoxic activity against AGS with IC_50_ 4.9 and 4.97 μΜ, respectively. The stronger effect on the MCF-7 line was also **88** with IC_50_ 0.17 μΜ. Against MCF-7, compound **83** presented an IC_50_ = 128 μΜ higher than the pyridine derivatives. Docking studies were consistent with these results. In general, hydrogen bonds and hydrophobic interactions are essential for inhibitory activity. Compounds **88** and **101** were the ones that possessed the highest free energy binding value of each series, −7.67 and −9.52 kcal/mol, respectively, meaning that they strongly bind to Eg5. For compound **88**, this can be attributed to the presence of H-bonds between NH- of the derivative and Glu116 of Eg5 stabilizing the enzyme, whereas for compound **101**, to the formation of hydrogen bonds with Glu118 and Glu117.

**Figure 16:**
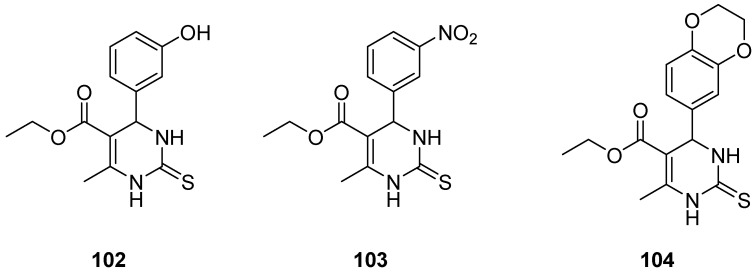
Compounds synthesized by Hernández et al. [8]. The most efficient is structure **103**.

Hernández et al. [8] synthesized a series of molecules that are both Eg5 and L-type calcium channel inhibitors (Figure 16). The latter participated in processes like metastasis, angiogenesis, and avoiding apoptosis and are being overexpressed in cancer cells. Regarding their vasorelaxing ability, three compounds were the most potent with IC_50_ ranging from 1.2 to 16 μΜ and compound **103** being stronger than the standard drug nifedipine. The latter one seems to affect the influx of Ca^2+^ rather than their intracellular level based on an assay that studied the contractions caused by CaCl_2_. Concerning their anticancer activity, the cytotoxicity of the compounds against A-549 and MCF-7 cancer cell lines was evaluated. Again, compound **103** was the most effective one with IC_50_ 44.9 and 32.2 μΜ for A-549 and MCF-7, respectively, presenting better results than the reference compound monastrol. When monastrol inhibits Eg5, the creation of monopolar spindle in the cell is a characteristic phenomenon that eventually leads to cell death. Thus, it was studied whether this phenomenon would occur or not in order to test if these molecules can inhibit Eg5. Compounds **103** and **104** were observed to increase the amount of monopolar spindle mitosis. It was impressive due to the fact that, in the presence of **103**, this phenomenon was 2,5-fold more intense than the negative control.

**Figure 17:**
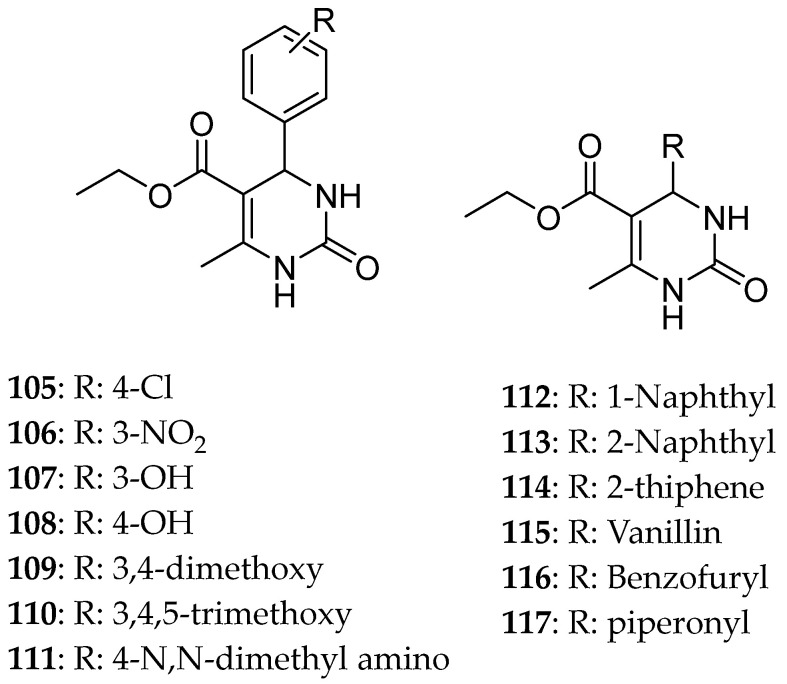
Dihydropyrimidines synthesized by Khanum et al. [16].

**Figure 18:**
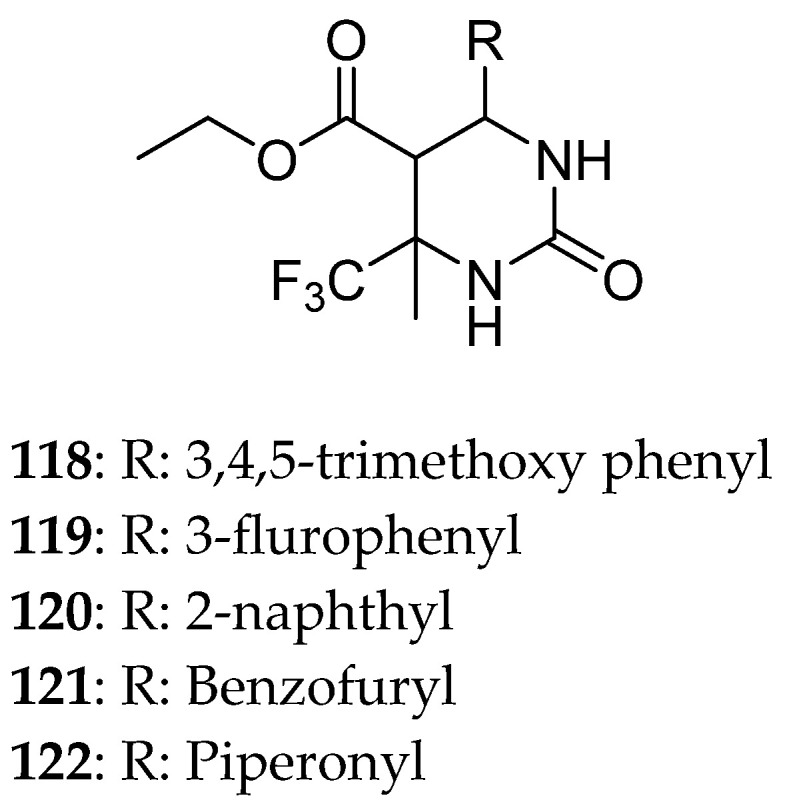
Trihydropyrimidines synthesized by Khanum et al. [16].

Khanum et al. [16] used SiO_2_ as a catalyst and produced a set of dihydropyrimidines (Figure 17) and a set of trihydropyrimidines (Figure 18) in order to discover the best and stereoselective procedure to synthesize DHPMs. All molecules follow Lipinski’s rule in which five possessed good absorption, four DHPMs can penetrate the BBB, while the others have a proper intestinal absorption. Compound **120** showed potential as an orally administrating agent with physicochemical characteristics similar to the standard drugs monastrol and piperastrol. These two compounds are well known for their ability to inhibit Eg5 and thus being cytotoxic to cancer cells. This compound was also tested for its cytotoxicity. Again, monastrol and piperastrol were used as reference compounds. Compound **120′**s anticancer ability was quite promising, with IC_50_ of 15.27 and 10.87 μg/mL against MCF-7 and HT-29, respectively, significantly better than monastrol and a little weaker than piperastrol. In addition to its physicochemical characteristic, it can be a promising drug candidate.

### 2.4. Proline and Cyclized Cysteine Like DHPM

**Figure 19:**
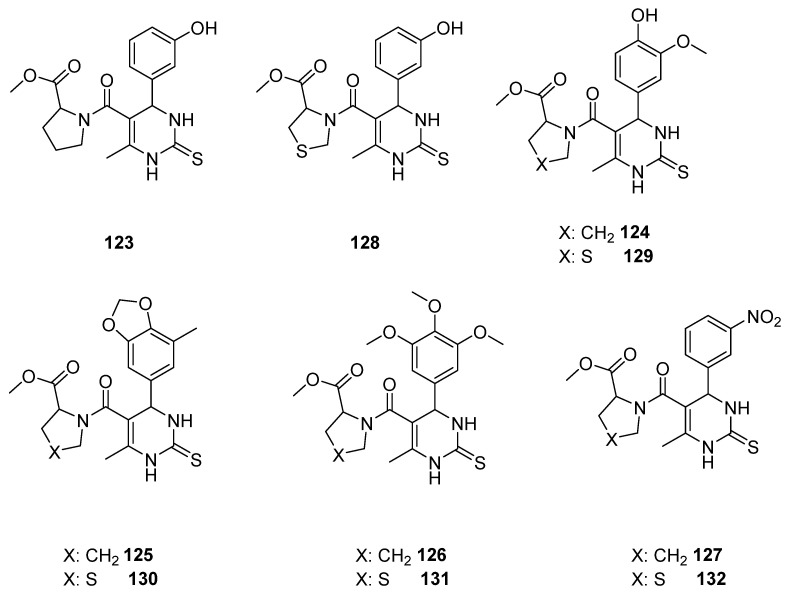
Proline and cyclized cysteine DHPM derivatives that Malik et al. synthesized [17].

Malik et al. [17], after highlighting the overexpression of Eg5 to cancer cells and its importance as a target of anticancer agents, modified monastrol, an already known Eg5 inhibitor, with amino acids cysteine and proline with an aim to produce powerful Eg5 inhibitors (Figure 19). Ten compounds with a substituted phenyl ring at C6 and with an amino acid connected to the ester of C5 were synthesized and tested as inhibitors of Eg5 and anticancer agents indirectly by studying their cytotoxic effect on cancer cell lines. Six of them were studied for their inhibition against 60 different cancer cell lines, whereas the other three were tested on a few specific ones. The majority of the compounds possessed cytotoxicity against CNS, renal cancer, and leukemia. Nevertheless, their ability is moderate to low. Regarding the most effective structure, every compound was effective toward a specific cell line. To elaborate, **131** was the one with the strongest antiproliferative effect concerning SNB-75 cancer cell line, while **125** was the most effective against renal RXF 393 cell line with 36.4% inhibition. Regarding leukemia, the most promising one is **130** with 53.3%, 43.2%, and 27.1% inhibition against SR, HL-60, and RPMI8226 cell lines, respectively. The other three compounds did not show an important anticancer effect; only **131** against colo-205 and **127** on A431 were stronger than monastrol but with moderate IC_50_ of 90 mg/mL. Regarding the molecular analysis, the interactions of **129** (cyclized cysteine moiety) and **124** (proline moiety) and monastrol with Eg5 were studied. Compound **124** develops more interactions with the enzyme forming four hydrogen bonds and hydrophobic interactions with fourteen amino acids with a binding energy of −8.11 kcal/mol. On the other hand, structure **129** hydrophobically interacts with thirteen amino acids and develops two hydrogen bonds with binding energy −7.48 kcal/mol. The two derivatives mentioned above were compared with monastrol, and it was concluded that monastrol presents remarkably less interactions with only one hydrogen bond and hydrophobic bonds with fewer amino acids. Hence, proline analogues bind stronger and present better values of binding energy to Eg5 compared with the cyclized cysteine ones. The pharmacokinetics of the molecules were also tested. Generally, all compounds have good cell permeability with **123–126** possessing good intestinal absorption. Lastly, for future studies, the potential of the structures to inhibit other targets was studied with **122** being a potential kinesin inhibitor.

## 3. Conclusions

In conclusion, this review reports research studies from 2018 to 2025 highlighting the importance of phenyl-substituted DHPM derivatives as promising anticancer agents. The results from the biological assays agreed with the docking ones. Their IC_50_s values of the biological assays are reported in Table 1. It is noteworthy that, in all cases where both thio- and oxo- derivatives were synthesized, the first ones consistently exhibited stronger antiproliferative activity than their oxo counterparts. Regarding the substituents of the phenyl ring, generally electron withdrawing groups in the para- or meta- position enhance the anticancer ability of a compound. For the phenyl ring at N3, the most effective structures like **8** and **16** have a halogen at the para- position of the ring. The most promising molecules with a phenyl ring to C6 are substituted at the meta- position with an electron withdrawing group or a bulky substituent. For example, compound **80** has a MeO group at the meta- and para- positions and **103** a NO_2_ group at the para- position, while **83** is substituted with thiophene, a bulky substituent. The replacement of the ethyl group of C5 with a phenyl ring like compounds **23** and **25** leads to quite promising structures. Substitution of this phenyl ring at the para- position with a halogen such as molecule **35** enhances the cytotoxic ability of the structure. Concerning the interactions with the active center of Eg5, hydrogen bonds and hydrophobic interactions were noticed between the most efficient structure and Eg5. Considering these results, it can be concluded that novel derivatives bearing these structural characteristics can be designed, synthesized, and studied for their cytotoxic activity aiming to obtain safe and efficient anticancer agents.

## Figures and Tables

**Table 1:** IC_50_ values of the compounds mentioned in the text.

Compound	IC_50_ (μΜ)									
	U-138	AGS	Hep-G2	MCF-7	Caco-2Cell	HeLa	T24	HepG3	A549	HT-29
**16**	18.52 ± 7.83									
**23**		9.9 ± 0.3	15.2 ± 0.4	40.5 ± 1.0						
**25**		23.8 ± 1.1	25.9 ± 7.2	94.2 ± 7.9						
**35**					65.80	75.59	103.21			
**57**				74.28 ± 8.81				74.01 ± 5.39	45.85 ± 1.54	
**58**								73.71 ± 4.26	43.97 ± 0.45	
**59**			124			187				
**60**			120			217				
**68**			73.71	65.54					43.97	
**80**		4.97								
**88**		4.9		0.17						
**83**				128						
**103**				32.2					44.9	
**120**				15.27						10.87

## Data Availability

No new data were created or analyzed in this study. Data sharing is not applicable to this article.

## Abbreviations

The following abbreviations are used in this manuscript:

A431	human epidermoid carcinoma cell line
A549	lung adenocarcinoma cell line
AGS	human gastric adenocarcinoma cell line
CaCo2	colorectal adenocarcinoma cell line
Cdk1	cyclin-dependent kinase 1
CNS	central nervous system
colo-205	colorectal adenocarcinoma cell line
DCF-DCA	dichlorodihydrofluorescein diacetate
DHPM	dihydropyrimidinones
DHPMT	dihydropyrimidinethiones
HeLa	cervical cancer cells
Hep-G2	human liver cancer cell line
HL-60	acute promyelocytic leukemia cell lines
Hsp90	Heat Shock Protein 90
MCF-7	human breast cancer cell line
RPMI8226	human multiple myeloma cell line
SR	human lymphoma cell line
T24	cell carcinoma of the bladder
